# Maternal testosterone affects offspring telomerase activity in a long‐lived seabird

**DOI:** 10.1002/ece3.9281

**Published:** 2022-09-12

**Authors:** Jose C. Noguera, Alberto Velando

**Affiliations:** ^1^ Grupo de Ecología Animal (GEA), Centro de Investigación Marina (CIM) Universidad de Vigo Vigo Spain

**Keywords:** androgens, cell signaling, growth, life‐history, yellow‐legged gull

## Abstract

Androgens are a group of steroid hormones that have long been proposed as a mechanism underpinning intergenerational plasticity. In birds, maternally allocated egg testosterone, one of the main androgens in vertebrates, affects a wide variety of offspring phenotypic traits but the mechanisms underlying this form of intergenerational plasticity are not yet well understood. Recent in vitro and animal model studies have shown that telomerase expression and activity are important targets of androgen signaling. The telomerase enzyme is known for its repair function on telomeres, the DNA–protein complexes at the ends of chromosomes that are involved in genomic integrity and cell aging. However, the role of maternal testosterone in influencing offspring telomerase levels in natural populations and its consequences on telomere length and potentially on offspring development is still unknown. Here, by experimentally modifying the level of egg testosterone in a natural population of yellow‐legged gull (*Larus michahellis*), we show that chicks hatched from testosterone‐treated eggs had higher average levels of telomerase and faster growth than controls during the first week of life. While testosterone‐treated chicks also tended to have longer telomeres than controls at hatching this difference disappeared by day 6 of age. Overall, our results suggest that maternal testosterone may have a potential adaptive value by promoting offspring growth and presumably telomerase levels, as this enzyme plays other important physiological functions (e.g., stress resistance, cell signaling, or tissue genesis) besides telomere lengthening. Nonetheless, our knowledge of the potential adaptive function of telomerase in natural populations is scarce and so the potential pathways linking maternal hormones, offspring telomerase, and fitness should be further investigated.

## Graphical abstract

Maternally‐allocated egg testosterone increased offspring telomerase activity and favoured a faster growth during postnatal development.

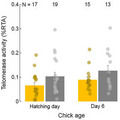

## 1 INTRODUCTION

In many organisms, the maternal phenotype has a significant influence on a variety of offspring traits through mechanisms other than maternal inheritance, a process that is usually known as ‘maternal effects’ (Mousseau et al., 2009; Wolf & Wade, 2009). Maternal effects are ubiquitous and can take many forms but, in oviparous species, one important pathway is through changes in egg composition, including the hormonal content (reviewed in Gil, 2003; Groothuis et al., 2019; Groothuis & Schwabl, 2008). These maternally allocated egg substances can be very important during embryonic development, as this is an especially sensitive time window of development where organisms undergo a fast rate of cell division, gene expression, and physiological changes that can ultimately affect the postnatal phenotype (Vaiserman et al., 2017, 2018). Indeed, maternal hormones have been shown to influence a wide variety of offspring phenotypic and life‐history traits such as growth and survival (von Engelhardt & Groothuis, 2011) but the mechanisms underpinning this form of intergenerational plasticity are not yet well understood.

In birds, it has been shown that mothers often transfer a variety of steroid hormones to the eggs that can have important effects on offspring growth and survival (Hayward & Wingfield, 2004; Tissier et al., 2014; von Engelhardt et al., 2004) and can even modulate stress reactivity and cellular aging (Haussmann et al., 2011; Hayward et al., 2006; Tissier et al., 2014). Notably, among these steroid hormones, many studies have focused on maternal androgens such as testosterone (Gil, 2003; Groothuis et al., 2019; Groothuis & Schwabl, 2008). Testosterone, one of the main androgens in vertebrates, accumulates in the egg yolk during follicle maturation (Schwabl, 1993) and its level often varies with laying order and/or in response to environmental or social factors (see e.g. Gil, 2003; Rubolini et al., 2011 and references therein). Importantly, higher levels of testosterone in the yolk have repeatedly been shown to increase offspring pre and postnatal development, competitive behavior or survival in different species (Eising et al., 2001; Navara et al., 2006; Rutkowska & Cichoń, 2006; Schwabl, 1996), effects that have led to suggest that testosterone allocation may be a form of adaptive maternal manipulation of offspring phenotype and performance (Groothuis et al., 2005; Groothuis & Schwabl, 2008).

An important route by which maternal testosterone may favor offspring performance is through changes in physiological pathways involved in the correct functioning of cells, tissues, and organs. In this regard, recent in vitro and animal model studies have shown that telomerase gene expression and activity are important targets of androgen signaling (Calado et al., 2009; Martínez & Blasco, 2017; Vasko et al., 2017), including testosterone (Bär et al., 2015; Nourbakhsh et al., 2010; Vieri et al., 2020). Telomerase is known to catalyze the elongation and maintenance of telomeres (Criscuolo et al., 2018; Smith et al., 2021). These noncoding nucleotide sequences cap the ends of chromosomes and in the absence of restoration, shorten with each cell division and in response to other factors such as oxidative stress (Reichert & Stier, 2017; Von Zglinicki, 2002). Notably, evidence indicates that, at least in large‐size mammals and several bird species, individuals with shorter telomeres or experiencing a greater loss of telomere length show lower short‐term survival and lifespan (e.g., Heidinger et al., 2012; Noguera et al., 2020; Wilbourn et al., 2018; but see also e.g. Tricola et al., 2018 and references therein).

Although often overlooked, telomerase has also been shown to have several other important physiological functions besides its role in telomere lengthening (reviewed in Ségal‐Bendirdjian & Geli, 2019; Thompson & Wong, 2020). For instance, evidence indicates that telomerase is implicated in redox homeostasis and stress resistance because under oxidative stress conditions, the catalytic subunit of telomerase (hTERT) is transported into the mitochondria to reduce ROS production and protect normal mitochondrial functions (Ahmed et al., 2008; Ale‐Agha et al., 2014; Rosen et al., 2020 and references therein). Moreover, telomerase plays an important role in cell signaling and gene expression, specifically interacting with key transcriptional factors essential for cell proliferation, tissue genesis, and immune regulation (see e.g. de Punder et al., 2019 and references therein) or even affects important epigenetic mechanisms such as DNA methylation (Yuan & Xu, 2019). Maternally allocated testosterone may therefore favor different important aspects of an individual's phenotype through its action on this enzyme.

In humans and large placental mammals, telomerase is highly active during embryonic development but later on, its activity is down‐regulated in most differentiated somatic cells after birth (Blackburn, 2005). However, the regulation of telomerase is not equal across taxa. Indeed, it has been shown that long‐lived bird species (e.g., seabirds) can maintain significant telomerase activity after hatching even in differentiated cells lacking proliferative capacity such as red blood cells (RBCs) (Haussmann et al., 2007; Noguera & Velando, 2021) and recent evidence suggests that this activity might be, at least partially, under the maternal influence (Noguera et al., 2020). Despite its recognized cellular functions and its potential role on individual fitness (Criscuolo et al., 2018), the study of telomerase in wild animal populations has largely been ignored and so, it is still unknown whether maternal testosterone may influence offspring telomerase levels and which are the consequences on offspring development.

To investigate to what extent maternally derived testosterone may influence offspring telomerase activity, telomere length, and postnatal development, we conducted a field experiment in a wild breeding population of yellow‐legged gull (*Larus michahellis*) and used testosterone injections to increase the level of egg testosterone within its natural physiological range. We predict that if yolk testosterone positively affects chick growth and the pathways involved in telomerase expression, chicks hatched from testosterone‐treated eggs should show faster growth and have a higher telomerase activity than sham‐injected (i.e., control) chicks. Although it is unlikely telomerase mediates telomere elongation long after RBCs are differentiated, in birds, hatchlings' RBCs are produced by the hematopoietic stem cells in the bone marrow during embryonic development (Sturkie, 2012) and RBCs have, on average, a longer lifespan than the time needed for gull embryos to develop (Rodnan et al., 1957). Thus, we further predict that our experimental treatment should lead to longer telomeres early after hatching, at least, whenever any postnatal variation in telomerase activity reflects changes that occur in other cell types and tissues during embryonic development.

Additionally, as postnatal telomerase may mediate the effects of growth on telomere dynamics and also play other physiological functions (i.e., different than telomere lengthening), we further explore the covariation pattern between offspring telomerase dynamics, telomere length, and postnatal growth. Here, we predict that the postnatal variation in telomerase activity and telomere length should be positively correlated whenever the variation in RBCs telomerase activity reflects that occurring in other tissues (e.g., bone marrow). Yet, if telomerase plays other functions besides telomere maintenance (e.g., improving stress resistance or tissue genesis), then this should be evidenced in a positive pattern of covariation between telomerase activity levels and postnatal growth.

## 2 MATERIAL AND METHODS

### 2.1 Study area and general procedures

We conducted the experiment from April to June 2021 in a gull breeding colony on Sálvora Island, Parque Nacional de las Illas Atlánticas de Galicia, Spain (42^o^28′N, 09^o^00′W). We visited the study area daily during the egg‐laying period and looked for nests with one laid egg. We then followed the nests daily to mark the following eggs and register their exact laying date. In this species, the modal clutch size is three eggs and so, only clutches of three eggs were used for the experiment. Once the third egg was laid, we collected the second eggs from 50 three‐egg clutches and transported them in an isothermal box to a field laboratory located outside the colony (<500 m). We focused on only one egg per clutch (i.e., second‐laid eggs) for practical and ethical reasons. First, we were only confident of the exact laying date of the second and third‐laid eggs and although within a clutch both eggs show higher levels of yolk testosterone than the first egg (Rubolini et al., 2011), the second egg has a substantially higher hatching success and postnatal survival than the third egg (Noguera & Velando, 2020). Yolk injections often result in a percentage of eggs failing to hatch (Noguera et al., 2011; Rubolini et al., 2006) and so, by focusing on the second eggs we aimed to minimize the number of eggs that had to be manipulated but maximizing our final sample size. We weighed the eggs in an electronic balance (±0.01 g) and randomly assigned them to either a “testosterone” (T) or “control” (C) treatment (*N* = 25 eggs in each group). In the testosterone group, we injected the egg yolk with a known amount of testosterone (Merck KGaA, Germany) dissolved in 20 μl of sterile sesame oil (for details of the egg injection protocol see Noguera et al., 2012). Because yolk testosterone varies according to egg size and laying order, we scaled the testosterone dose according to egg mass and laying order as previously described for this species (Parolini et al., 2017). The doses (ng) were as follows depending on the egg size class (g): 80–88 g: 74 ng, 89–92 g: 73 ng, 93–99 g: 81 ng. These doses of testosterone have previously been shown to increase the final yolk concentration by 1 SD of the concentration recorded in other colonies of the same species (Parolini et al., 2017; Parolini et al., 2019). In the control group, we injected the eggs with 20 μl of sesame oil using the same procedure as for the testosterone eggs. After the injections, the hole in the shell was sealed with a patch of hen eggshell previously sterilized as previously described (see e.g. Noguera et al., 2011). The eggs were then returned to their original nest and the whole clutch was then cross‐fostered between pairs of (experimental) nests that had the same laying date (±1 day).

We checked the nests twice a day, beginning 2 days before the estimated hatching date. At hatching, we marked all chicks with numbered leg flags for their identification. Nine eggs did not hatch (4T and 5C) and three more were found predated (1T and 2C), but hatching success did not differ between experimental groups. We blood sampled and measured all experimental chicks at two different ages: at hatching day and day six of age. Although close in time, the second sampling (day 6 of age) allowed us to assess whether or not any effect of our experimental treatment on telomerase activity and telomere length remained after hatching and related to any effect of our experimental treatment on postnatal growth (see e.g. Rubolini et al., 2006). We collected small blood samples (approx. 90 μl) from the wing vein and weighed them and measured them with a spring balance (±1 g) and a caliper (±0.001 mm), respectively. Blood samples were immediately transported to our field laboratory in a cooler filled with ice packs (i.e., within 1 hr after collection), centrifuged and the plasma and red blood cells (RBCs) fractions were stored in liquid nitrogen. Once in the laboratory, samples were stored at −80°C until the laboratory analyses were performed (within 2–3 weeks after the end of the field experiment).

### 2.2 Laboratory analyses

Telomerase activity in RBCs was measured using the quantitative telomeric repeat amplification protocol (Q‐TRAP) assay (Herbert et al., 2006), with some minor modifications described for this species (see Noguera et al., 2020 for further details). The telomerase activity of each sample was quantified based on the linear equation of the standard curve derived from a serially diluted positive control sample (*R* ^2^ > 0.99 in all cases) and the values were normalized to those of a positive reference sample and expressed as a percentage of relative telomerase activity (%RTA). Four samples (2T and 2C) were incorrectly labeled and therefore had to be excluded from the analyses. All samples were run in triplicate and the repeatability (ICC) of %RTA based on triplicates was 0.78, (*N* = 64, *p* < .001) and the interplate coefficient of variation based on one sample repeated over all plates was 5.6%.

Telomere length was measured in RBCs DNA samples using the same qPCR device as above and following the real‐time quantitative PCR assay described by Criscuolo et al. (2009) and adapted for yellow‐legged gull samples (Kim & Velando, 2015). The qPCR method “normalizes” the quantity of telomere product (T) to a single‐copy gene (S) to provide a mean telomere length for the cell population (T/S ratio). The yellow‐legged gull GAPDH gene was used as a single‐copy gene in all analyses and the efficiency of each amplicon (TEL and GAPDH) was estimated from the slopes of the amplification curves for each qPCR reaction using LinRegPCR software (range 80–82%) (Ruijter et al., 2009). All samples were run in triplicate and the repeatability (ICC) of the T/S values based on triplicates was 0.87 (*N* = 69, *p* < .001) and the interplate coefficient of variation based on one sample repeated over all plates was 5.5%.

Gull chicks were also sexed by molecular analysis using the primer sequences described by Fridolfsson and Ellegren (1999).

### 2.3 Statistical analyses

Firstly, we used generalized linear models (with binomial error distribution; GLM) or linear models (LM) to confirm that there were no initial between‐group differences in egg mass (LM), laying date (LM; in Julian), hatching success (GLM), or sex‐ratio (GLM). All of these initial models included the experimental treatment as a fixed factor.

Secondly, we used linear mixed‐effects models (LMMs) to test the postnatal effect of the testosterone treatment on chick telomerase activity, telomere length, body mass, and tarsus length measured at hatching day and day 6 of age. The models included the experimental treatment (i.e., C or T), chick age (two levels; day 0 or 6 of age) and their two‐way interaction as fixed factors, and chick identity (ID) as a random factor. In all models, we also controlled for chick sex and egg mass but we did not include additional interactions for which we had no a priori predictions to reduce the complexity of the models. Additionally, we further performed complementary correlation analyses to explore whether any change in telomere length and growth rates was related to an age‐related change in telomerase activity.

All analyses were conducted using IBM SPSS Statistics 26 for Windows (IBM Corp.). In all models, Satterthwaite's degrees of freedom were used and when needed, post hoc tests were performed and their FDR‐ adjusted and unadjusted P‐values were reported. In all models, nonsignificant interactions were removed as recommended (Engqvist, 2005) and the proportion of variance explained for mixed models was assessed by calculating the marginal and conditional *R* ^2^ (i.e., *R* ^2^_m_ and *R* ^2^c; Nakagawa & Schielzeth, 2013). Normality and homoscedasticity assumptions were checked in all models. Slight differences in sample sizes in some analyses reflect missing values due to the death or loss of chicks. Unless specified, data are presented as means ± standard errors (SE), and the significance level was set at *p* = .05.

## 3 RESULTS

Our preliminary analyses showed that neither egg mass (*F*_1,48_ = 0.192, *p* = .663) nor laying date (*F*_1,48_ = 0.719, *p* = .401), hatching success (Wald‐χ^2^ = 0.194, df = 1, *p* = .659) or sex‐ratio (Wald‐χ^2^ = 0.117, df = 1, *p* = .732) differed between testosterone‐treated and control eggs.

**FIGURE 1:**
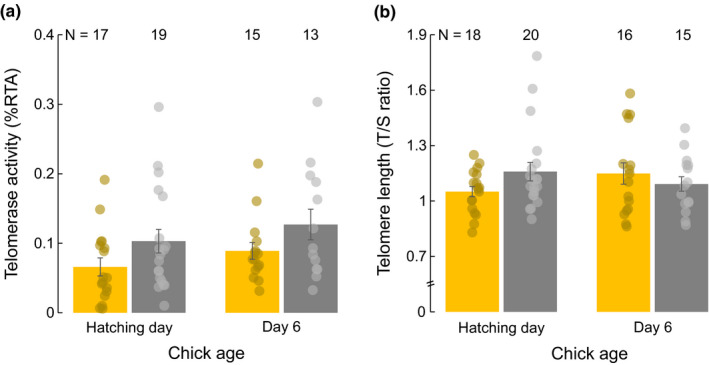
(a) Telomerase activity and (b) telomere length at hatching day and day six of age in gull chicks from control (yellow bars) and testosterone‐treated (gray bars) eggs. Data show mean ± SEM.

Early postnatal levels of telomerase activity differed between experimental groups (Table 1); on average, chicks hatched from testosterone‐treated eggs showed higher telomerase activity than control chicks during their first 6 days of life (treatment × age: *F*_1,32.30_ = 0.001, *p* = .986; Table 1; Figure 1a). We also found that telomere length during the early postnatal period varied between experimental groups, as evidenced by the significant interaction between the experimental treatment and age (Table 1; Figure 1b). Thus, chicks hatched from testosterone‐treated eggs tended to have longer telomeres than control chicks at hatching (unadjusted *p* = .07, FDR‐adjusted *p* = .26) but afterward, telomere length followed a contrasting trend in both groups which led to no clear differences between groups at day 6 of age (unadjusted *p* = .47, FDR‐adjusted *p* = .60). The rest of the variables included in the above models were nonsignificant (Table 1). Our supplementary analyses indicated that the observed postnatal variation in telomere length was unrelated to the change in telomerase activity (Pearson's correlation coefficient: *r* = 0.06, *p* = .750).

**TABLE 1:** Summary of LMMs for the effects of testosterone treatment and covariates on telomerase activity, telomere length, body mass, and tarsus length of yellow‐legged gull chicks between hatching day (day 0) and day 6 of age.

Dependent variable	Source of variation	Estimate	*F*‐value	df_*n*,*d*_	*p*‐value
Telomerase activity	Intercept	0.113			
*R* ^2^_(M)_ = 0.103	Treatment (C)	−0.037	4.364	1,33.32	**.043**
*R* ^2^_(c)_ = 0.317	Age (day 0)	−0.022	2.402	1,33.30	.131
	Sex (female)	−0.016	0.763	1,33.91	.388
	Egg mass	2.25e^−4^	0.024	1,32.35	.878
	*Random effect*	*Variance*			
	Chick ID	0.001			
	Residual	0.003			
Telomere length	Intercept	1.074			
*R* ^2^_(M)_ = 0.058	Treatment (C)	0.049	0.296	1,33.67	.590
*R* ^2^_(c)_ = 0.461	Age (day 0)	0.064	0.178	1,31.44	.676
	Sex (female)	−0.033	0.341	1,34.03	.563
	Egg mass	0.004	0.009	1,33.02	.926
	Treatment × age	−0.159	5.056	1,31.61	**.032**
	*Random effect*	*Variance*			
	Chick ID	0.016			
	Residual	0.020			
Body mass	Intercept	0.918			
*R* ^2^_(M)_ = 0.713	Treatment (C)	−4.305	4.921	1,35.95	**.033**
*R* ^2^_(c)_ = 0.744	Age (day 0)	−20.264	132.829	1,37.46	**<.001**
	Sex (female)	2.332	1.353	1,36.34	.252
	Egg mass	0.9132	33.890	1,35.27	**<.001**
	*Random effect*	*Variance*			
	Chick ID	6.427			
	Residual	52.661			
Tarsus length	Intercept	22.352			
*R* ^2^_(M)_ = 0.777	Treatment (C)	−1.459	7.170	1,36.21	**.011**
*R* ^2^_(c)_ = 0.835	Age (day 0)	−4.560	270.154	1,35.72	**<.001**
	Sex (female)	0.336	1.144	1,36.52	.292
	Egg mass	0.085	12.172	1,35.49	**<.001**
	Treatment × age	1.287	7.306	1,35.72	**.010**
	*Random effect*	*Variance*			
	Chick ID	0.259			
	Residual	1.126			

*Note*: Significant results (*p* < .05) are highlighted in bold.

**FIGURE 2:**
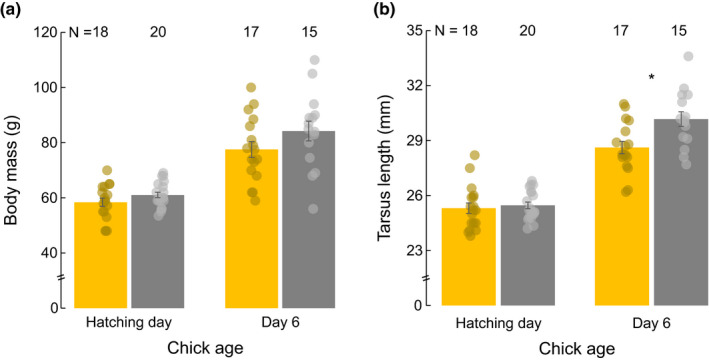
(a) Body mass and (b) tarsus length at hatching day and day six of age in gull chicks from control (yellow bars) and testosterone‐treated eggs (gray bars). Data show mean ± SEM significant post hoc comparisons between experimental groups within each age class are denoted by ‘*’.

Testosterone treatment also affected chick body mass and tarsus length during the early postnatal period, although in the case of tarsus length there was a significant interaction between the experimental treatment and age (Table 1). Thus, chicks hatched from testosterone‐treated eggs were, on average, heavier than the controls during the first 6 days of age (treatment × age: *F*_1,36.42_ = 0.60, *p* = .389; Table 1; Figure 2a) and also attained a bigger structural size on day six of age (unadjusted *p* < .001, FDR‐adjusted *p* = .001; Table 1; Figure 1b). Chick body mass and tarsus length did not differ between sexes but were positively correlated to egg mass (Table 1). Our complimentary analysis revealed no pattern of covariation between the change in telomerase activity and postnatal growth in body mass (Pearson's correlation coefficient: *r* = 0.247, *p* = .206) or tarsus length (Pearson's correlation coefficient: *r* = 0.260, *p* = .181).

## 4 DISCUSSION

Here, we show that maternal testosterone can induce multiple effects affecting various offspring traits, including early postnatal telomerase activity, telomere length, body mass, and tarsus length. During the first 6 days after hatching, the chicks of testosterone‐treated eggs had, on average, higher levels of telomerase. Such an effect, however, was not mirrored in postnatal telomere length; while testosterone‐treated chicks tended to have a longer telomere length than controls at hatching, by 6 days of age telomere length was similar in both experimental groups. Moreover, our additional analyses showed that the early postnatal variation in telomerase activity did not correlate with the change in telomere length, suggesting that changes in RBCs telomerase did not mirror processes governing changes in RBCs telomere length. Yolk testosterone also favored the postnatal growth of the gull chicks but this effect was neither related to the observed changes in telomerase activity. While faster growth during early postnatal life may report some potential benefits to the young birds, the benefits of having increased telomerase activity levels still need to be confirmed in future studies.

Our results show that yolk testosterone had a positive effect on early postnatal telomerase activity. This interesting result supports previous evidence from biomedical studies indicating an active role of androgens in upregulating telomerase activity both in vivo (Bär et al., 2015) and in vitro (Nourbakhsh et al., 2010; Vieri et al., 2020). Although the exact mechanisms by which maternal testosterone stimulates offspring telomerase activity early after hatching is still unknown, a possibility is that increased yolk testosterone triggered the prenatal and early post‐natal upregulation of the hTERT gene (Bär et al., 2015; Nourbakhsh et al., 2010), thereby favoring the catalytic subunit and the key determinant of telomerase activity (Cong et al., 2002). Furthermore, higher testosterone levels may have increased metabolic activity (Tobler et al., 2007) and so, the production of reactive oxygen species and/or DNA damage (Treidel et al., 2013), molecules that activate signaling pathways involved in telomerase upregulation (Fouquerel et al., 2016; Lee et al., 2017). Yet, the latter possibility seems less likely, as increased levels of yolk testosterone do not appear to favor the production of pro‐oxidant molecules during the first days after hatching in this species (Noguera et al., 2011; Parolini et al., 2018). Irrespective of the mechanism, our results suggest that maternal androgens may have a programming effect on offspring telomerase activity early after hatching.

The testosterone treatment in interaction with age also affected early postnatal telomere length during the first 6 days after hatching. However, in contrast to our expectations, the effect of our treatment on offspring postnatal telomere length did not match with that observed in telomerase. This was further corroborated by the lack of covariation between postnatal variation in telomerase and telomere length. These results are, however, not surprising, taking into account that the repair capacity of telomerase takes place during cell division (Armstrong & Tomita, 2017; Criscuolo et al., 2018) and bird RBCs do not longer divide once differentiated. Thus, our results suggest that postnatal telomere length is likely to be modulated by mechanisms other than telomerase expressed in cells (and tissues) with low or null proliferative potential. Indeed, this may explain why telomerase activity and telomere length do not always positively covary during the postnatal growth period in this species (Noguera & Velando, 2021). Although we do not know the causes of the contrasting trends in telomere length between the experimental groups, it is plausible that they were influenced by an effect of our hormonal treatment on other (unmeasured) phenotypic traits. For instance, if increased yolk testosterone promoted a more active and competitive chick phenotype (e.g., increasing begging behavior and/or activity levels; Gil, 2003), these behavioral changes might have increased the demands for some important antioxidants (Noguera et al., 2010) and favored the observed decline, although not significant, of telomere length with age (Kim & Velando, 2015). In any case, our results suggest that, at least in differentiated somatic cells like RBCs, telomerase and telomere length are probably not as closely coupled as expected and probably deeply influenced by other (unmeasured) environmental factors.

Our results also show that our testosterone treatment favored offspring postnatal growth, an effect that is in agreement with previous studies in birds (see e.g. Gil, 2003; Groothuis et al., 2005 and references therein), including this and other seagull species (Eising et al., 2001; Parolini et al., 2017). The boosting effect of egg testosterone on early postnatal growth may be the result of the well‐known anabolic effects that androgens have on muscles and bones in birds (Meyer, 2001; Navara & Mendonça, 2008), tissues with a high density of androgen receptors (Compston, 2001; Corvol et al., 1992). A higher level of egg testosterone may have stimulated chick body mass and bone growth via different pathways, including the secretion of growth factors, protein synthesis, or increasing mineral absorption (see e.g. Meyer, 2001; Navara & Mendonça, 2008; Urban, 2011; West & Phillips, 2010). Postnatal growth did not correlate to changes in telomerase activity, probably indicating that any effect of telomerase on chick postnatal development was minor, at least during the first days after hatching. However, we cannot discard the possibility that increased telomerase activity might play a role later on when energetic demands substantially increase to sustain the maximal growth rate.

While attaining more body mass and a bigger structural size might involve some fitness benefits for testosterone‐treated chicks, a faster postnatal growth may also have some potential costs. For instance, in this and other bird species, faster postnatal growth has been associated with increased oxidative stress levels during development (Kim et al., 2011; Metcalfe & Alonso‐Alvarez, 2010). High levels of oxidative stress may negatively affect chick health status and survival by either directly reducing the correct functioning of tissues and organs (Rahman et al., 2012) or by accelerating the rate of telomere shortening (Monaghan & Ozanne, 2018; Salmón et al., 2021). Yet, as the change in postnatal telomere length did not correlate with postnatal growth, the impact of faster growth on postnatal telomere length was probably minor, at least, during the first days after hatching.

Egg testosterone increased telomerase activity early after hatching but such an effect did not relate to telomere length or growth, suggesting that telomerase may play other functions. In this regard, recent studies have shown that telomerase plays a key antioxidant function in the mitochondria, especially under oxidative conditions (Rosen et al., 2020). As yellow‐legged gull chicks are often exposed to increased levels of oxidative stress due to different environmental and social factors (Noguera et al., 2010, 2017; Noguera & Velando, 2020) and increased levels of oxidative stress early after hatching can compromise future survival (Noguera et al., 2012), it might be possible that gull chicks benefit of maintaining higher levels of telomerase early after hatching. Additionally, as telomerase can also enhance T‐cells proliferation (Qian et al., 2014), any maternally mediated increase of telomerase expression may potentially increase offspring survival by favoring early immune responses (Norris & Evans, 2000). Yet, our understanding of the non‐canonical functions of telomerase (i.e., beyond telomere maintenance) in natural populations and their implications in wild animal populations is still in its infancy and so, the potential adaptive value of any maternally mediated increase of telomerase still needs to be confirmed.

In conclusion, our findings demonstrate that maternally allocated egg testosterone has multiple effects on the offspring, increasing telomerase activity and growth, and affecting postnatal telomere length. As more evidence emerges suggesting that telomerase may have a role in cell mitochondrial functioning or signaling pathways, our results provide a new mechanism for maternal testosterone to act as a tool for adjusting offspring phenotypic development and life‐history trajectories. Nonetheless, our knowledge of the potential adaptive value of this form of maternal programming of telomerase activity is still largely lacking and deserves further investigation. Future experimental studies should investigate whether the observed changes in telomere maintenance mechanisms vary in response to other maternally allocated egg components or the prevailing environmental conditions, and relates to important aspects of offspring fitness.

## AUTHOR CONTRIBUTIONS

**Jose Carlos Noguera:** Conceptualization (lead); data curation (lead); formal analysis (equal); investigation (lead); writing – original draft (equal); writing – review and editing (equal). **Alberto Velando:** Formal analysis (equal); funding acquisition (lead); project administration (lead); resources (lead); writing – original draft (equal); writing – review and editing (equal).

## CONFLICT OF INTEREST

The authors declare no competing interests.

## Data Availability

All data needed to evaluate the conclusions of the study are presented in the paper and/or the Supplemental Information. Raw data can also be found in the Figshare digital repository, https://doi.org/10.6084/m9.figshare.19890874.
